# Coronavirus disease 2019 is delaying the diagnosis and management of chest pain, acute coronary syndromes, myocarditis and heart failure

**DOI:** 10.2217/fca-2020-0088

**Published:** 2020-07-01

**Authors:** Bhurint Siripanthong, Thomas C Hanff, Michael G Levin, Mahesh K Vidula, Mohammed Y Khanji, Saman Nazarian, Choudhary Anwar A Chahal

**Affiliations:** ^1^School of Clinical Medicine, University of Cambridge, Cambridge, CB2 0SP, UK; ^2^Division of Cardiovascular Medicine, Hospital of The University of Pennsylvania, PA 19104, USA; ^3^Mayo Clinic Rochester, MN 55905, United States; ^4^Royal Papworth Hospital, NHS Trust, Cambridge, CB2 0AY, UK; ^5^Department of Cardiology, Newham University Hospital and Barts Heart Centre, Barts Health NHS Trust, London, E13 8SL, UK

**Keywords:** acute coronary syndrome, myocardial injury, percutaneous coronary intervention, SARS-CoV-2, sepsis-related cardiomyopathy, ST-elevation myocardial infarction, stress-induced cardiomyopathy, thromboembolism

Coronavirus disease 2019 (COVID-19) was first identified in December 2019, yet within months it had spread to pandemic levels with critical global health implications to the clinical practice of all specialties. In cardiology, COVID-19 has imposed an unfortunate conundrum: the disease is significantly associated with death in patients with pre-existing cardiovascular disease, [1,2] and yet hospital admissions and certain cardiac procedures have significantly declined compared with prior years [3–5]. This creates a potentially multiplicative problem if COVID-19 and its etiologic virus, the severe acute respiratory syndrome coronavirus-2 (SARS-CoV-2), directly or indirectly cause increased cardiac damage while patients with cardiac disease are simultaneously undertreated for pre-existing or *de novo* illness. Here, we assess how COVID-19 may have influenced this apparent decline in cardiac care. We also consider how clinicians can optimize the diagnostic process to provide appropriate and timely patient care in the setting of increased uncertainty regarding the etiology of acute cardiovascular disease. This process is dynamic and will necessitate adaptive strategies over time, particularly as many hospitals, states and countries transition slowly toward the re-escalation of standard operations while being mindful of COVID-19 resurgence.

In spite of numerous mechanisms that may increase the likelihood or severity of cardiovascular disease in COVID-19, many centers have thus far reported a decline in the number of patients admitted for the acute coronary syndrome (ACS). A recent report from the British Heart Foundation revealed that the patient footfall seeking medical attention for myocardial infarction, which is defined as myocardial cell death due to prolonged ischemia, was essentially halved in March 2020, compared with the same period a year ago in England [6]. This worrying trend is similarly observed in other countries severely affected by COVID-19, including the USA [5] and Spain; [7] the latter saw, among its 81 centers, the numbers of percutaneous coronary intervention (PCI) (48% decrease), cardiac structural interventions (81% decrease) and diagnostic procedures (56% decrease) dropped, significantly. Several societal, health system and infection-control issues have likely contributed to the decreased frequency of timely cardiac diagnoses during this pandemic. The morbidity and mortality associated with COVID-19 understandably begets fear and concerns in the general population. Coupled with government public health policies recommending patients to avoid the hospital unless suffering from severe COVID-19 symptoms or medical emergencies, rates of hospitalization have overall decreased. While a *bona fide* reduction in the incidence of ACS in the general population due to the imposed lockdown is possible, such as reduced work-related stress, decreased physical stressors and possible environmental factors such as air pollution reduction, we suspect these factors to be minor. Conversely, pandemic-induced stress and economic stress from unemployment can negatively impact cardiovascular health [8].

What is far more concerning, however, is that patients may avoid seeking treatment despite serious cardiovascular symptoms due to fear of nosocomial COVID-19 infection or concerns of adding to the overflowing workload at hospitals. Furthermore, the way in which certain centers approach the diagnosis of ACS or myocardial infarction in COVID-19 patients may have contributed to the decline in the apparent incidence of ischemic heart disease. For instance, some have advocated measuring cardiac enzymes only when the patients exhibit specific cardiac symptoms including chest pain, dyspnea and palpitations [9]. This is based on observational studies which showed that elevated cardiac biomarkers including cardiac troponin and NT-proBNP are common among the patients hospitalized for COVID-19, with up to 27.8% of the patients reported to have raised troponins [1]. However, a significant proportion of these patients were shown to have no underlying ACS or other cardiac pathology [9], prompting the notion that COVID-19 might predispose to a nonspecific cardiac enzyme leak which does not necessarily indicate Type 2 myocardial infarction [10]. While there is some rationale to limiting troponin tests to COVID-19 patients with overt cardiac symptoms, especially in the setting of over-burdened clinical resources and the potential for unnecessary staff viral exposure, restricted cardiac enzyme testing could potentially miss true ACS and other cardiac pathology when relatively minor cardiac symptoms are overshadowed by more severe respiratory symptoms. Some patients may also have silent infarctions, which are common in diabetics and in the elderly [11], particularly when delirium is present. Recognizing the population-prevalence of COVID-19 can help clinicians establish the pretest likelihood that cardiovascular symptoms or biomarker elevations are related to traditional cardiovascular diseases, or represent complications from COVID-19 infection, and inform testing and treatment strategies. This is particularly important as the hitherto evidence suggests that COVID-19 patients with raised cardiac biomarkers or pre-existing cardiovascular comorbidities have a worse prognosis, and they require higher utilization of intensive care and mechanical ventilation [2].

In spite of the broad etiologic differential for cardiac enzyme elevation, ACS remains highly likely and should always be considered, given the critical implications of a timely diagnosis. Several aspects of COVID-19 may increase the likelihood of ACS above baseline risk, both via *de novo* thrombotic events and the precipitation of subclinical coronary artery disease. The presence of acute respiratory distress syndrome and hypoxemia could exacerbate the oxygen demand-supply mismatch that leads to a type 2 myocardial infarction. For those with pre-existing atherosclerotic plaque, SARS-CoV-2 may predispose to increased atherosclerotic plaque instability and coagulopathy, which are increased in the setting of cytokine storm and may manifest as thromboembolic occlusion of the coronary artery [12]. Similar mechanisms have been proposed to explain the increased incidence of myocardial infarction following influenza and other respiratory infections [13]. Furthermore, large vessel pulmonary embolism, pulmonary microvascular thrombus and hypoxic vasoconstriction may lead to many incidences of sudden right ventricular dysfunction and *cor pulmonale*, which can also result in cardiac damage and cardiac enzyme release. These latter diagnoses are corroborated by recent autopsy series of COVID-19 patients demonstrating that venous thromboembolism and pulmonary embolism are frequent and often considered the immediate cause of death [14]. While the suspected pro-thromboembolic sequela of COVID-19 are not unique among other etiologies of sepsis and viral pneumonia [15], the rate of thromboembolic events appears to be increased. Compared with patients who died from H1N1 influenza, a recent autopsy series found that patients who died from COVID-19 had a ninefold increased incidence of alveolar capillary microthrombi (p < 0.001) [16].

The differential etiology for cardiac enzyme elevation is broad, even in the absence of viral infection, and this uncertainty is further compounded by COVID-19. Up to 40% of COVID-19 patients with ST-elevation myocardial infarction (STEMI) taken to diagnostic coronary angiogram have had no identifiable culprit occlusion [17]. Thus, while the majority of STEMI events remain related to coronary occlusion, other etiologies should be simultaneously considered, including myocarditis, sepsis-related cardiomyopathy, stress-induced (Takotsubo) cardiomyopathy, heart failure exacerbation with increased ventricular wall strain, endotheliitis with cardiac microvascular dysfunction and direct viral cardiomyocyte damage [10,12,18]. These require the use of multimodality imaging and in some cases eodomyocardial biopsy for accurate diagnosis – these are not being utilized due in part to over-burdened healthcare systems and in attempts to minimize spread to other areas of the hospital. Thus, this introduces inaccuracies and bias, potentially invalidating findings. Recent small necropsy studies have increased a concern for the latter two diagnoses (microvascular dysfunction and direct cardiomyocyte damage) due to the identification of viral inclusion bodies within endothelial cells, sequestered mononuclear and polymorphonuclear cellular infiltration of the endothelium, and evidence of endothelial cell apoptosis [19]. This is corroborated by a relative paucity of myocarditis, which has only occasionally been identified in patients with elevated cardiac enzymes. However, both the necropsy and imaging studies are not systematic and prone to selection bias, and are likely underestimates [18].

Given the importance of timely management of myocardial infarction in COVID-19 patients, clinicians must be able to distinguish true ACS from ACS mimics among these patients. The mainstay of ACS diagnosis is a raised troponin (above 99th percentile of the upper reference range limit) and at least one other sign including ECG changes such as ST-elevation, T-wave changes, *de novo* left bundle branch block and pathological Q waves; chest pain consistent with ischemia; imaging evidence showing new regional wall abnormalities or infarct; and angiographic evidence of coronary occlusion [20]. Patients with diagnosed STEMI must be offered PCI or, when the former is not promptly available, fibrinolysis. While COVID-19 patients should have a low threshold to trigger the troponin tests, initiating the primary PCI protocol for the COVID-19 patients based on raised troponins alone or troponins with less specific ECG findings could overwhelm the department’s capacity, given how common elevated cardiac biomarkers are among the infected. Moreover, in most STEMI mimics, including myocarditis or direct viral cardiomyocyte damage, stress-induced cardiomyopathy, there is no ischemic damage to the heart and the standard PCI protocol would be of little clinical benefit, yet this may expose both the patients and health professionals to unnecessary procedural and infection risk. In these cases, cardiac imaging could help refine the diagnosis, if clinicians triaging the decision for coronary angiography are uncertain of the presence of ACS, or the severity of illness warranting potential intervention. Hand-held, point-of-care echocardiography could be readily deployed to assess for the structural abnormalities of the heart walls and systolic function while patients undergoing high-resolution computed tomography for the assessment of respiratory pathology could be offered an additional contrast-enhanced cardiac computed tomography that would add little time and no additional contamination risk. Cardiac magnetic resonance may also be informative regarding cardiac involvement even in the absence of interstitial pneumonia [21]. These tools have the potential to streamline the diagnosis of concomitant cardiovascular disease. At last, if a patient with troponin elevation and acutely depressed myocardial contractility has just undergone coronary angiography excluding occlusive coronary artery disease, performing endomyocardial biopsy may be of benefit. This is because the suspicion of fulminant myocarditis is high, and the endomyocardial biopsy can help identify active cardiac infection and aid in the decision to initiate immunosuppressive agents [22].

Currently, the prevalence of myocardial infarction and its mimics among COVID-19 patients remains unclear. Certain initiatives have been made to study the epidemiology of ischemic heart disease in the SARS-CoV-2 infection; [23] and other diagnoses should be considered in order to recognize them and better understand their pathophysiology. It is hoped that these efforts will shed some light into the prevalence of the cardiac involvement and the longer term complications that might ensue. This valuable insight will undoubtedly help refine our strategies to manage myocardial infarction at an institutional level and inform future guideline recommendations on optimum preparation for any further waves of COVID-19 infection that front-line health professionals may face.
